# The transformative potential of mRNA vaccines for glioblastoma and human cancer: technological advances and translation to clinical trials

**DOI:** 10.3389/fonc.2024.1454370

**Published:** 2024-09-27

**Authors:** Iulia Tapescu, Peter J. Madsen, Pedro R. Lowenstein, Maria G. Castro, Stephen J. Bagley, Yi Fan, Steven Brem

**Affiliations:** ^1^ Perelman School of Medicine, University of Pennsylvania, Philadelphia, PA, United States; ^2^ Division of Neurosurgery, Children’s Hospital of Philadelphia, Philadelphia, PA, United States; ^3^ Department of Neurosurgery, University of Pennsylvania, Philadelphia, PA, United States; ^4^ Department of Neurosurgery, The University of Michigan, Ann Arbor, MI, United States; ^5^ Department of Cell and Developmental Biology, The University of Michigan, Ann Arbor, MI, United States; ^6^ Department of Biomedical Engineering, The University of Michigan, Ann Arbor, MI, United States; ^7^ Division of Hematology/Oncology, Department of Medicine, University of Pennsylvania, Philadelphia, PA, United States; ^8^ Glioblastoma Translational Center of Excellence, Abramson Cancer Center, University of Pennsylvania, Philadelphia, PA, United States; ^9^ Department of Radiation Oncology, University of Pennsylvania, Philadelphia, PA, United States

**Keywords:** brain tumor, clinical trial, glioma, glioblastoma, immunotherapy, immuno-oncology, mRNA, vaccine

## Abstract

Originally devised for cancer control, mRNA vaccines have risen to the forefront of medicine as effective instruments for control of infectious disease, notably their pivotal role in combating the COVID-19 pandemic. This review focuses on fundamental aspects of the development of mRNA vaccines, e.g., tumor antigens, vector design, and precise delivery methodologies, – highlighting key technological advances. The recent, promising success of personalized mRNA vaccines against pancreatic cancer and melanoma illustrates the potential value for other intractable, immunologically resistant, solid tumors, such as glioblastoma, as well as the potential for synergies with a combinatorial, immunotherapeutic approach. The impact and progress in human cancer, including pancreatic cancer, head and neck cancer, bladder cancer are reviewed, as are lessons learned from first-in-human CAR-T cell, DNA and dendritic cell vaccines targeting glioblastoma. Going forward, a roadmap is provided for the transformative potential of mRNA vaccines to advance cancer immunotherapy, with a particular focus on the opportunities and challenges of glioblastoma. The current landscape of glioblastoma immunotherapy and gene therapy is reviewed with an eye to combinatorial approaches harnessing RNA science. Preliminary preclinical and clinical data supports the concept that mRNA vaccines could be a viable, novel approach to prolong survival in patients with glioblastoma.

## 1 Introduction

In the realm of medical breakthroughs, few innovations have sparked as much excitement and promise as the advent of messenger ribonucleic acid (mRNA) vaccines (1–4), reflected in the award of the 2023 Nobel Prize in Physiology or Medicine to Katalin Karikó and Drew Weissman for their foundational discoveries of the mRNA vaccine platform (5). Importantly, the mRNA vaccine platform was originally adapted as a tool in the fight against cancer (6, 7). Sahin et al. noted a synergistic effect of mRNA vaccine with immune checkpoint blockade in patients with melanoma; antitumor responses were noted, paradoxically, in patients whose tumors had a low mutational burden, suggesting that mRNA vaccines could be effective in tumors (such as glioblastomas) with a low mutational burden (7).

The mRNA vaccine platform, however, emerged as a transformative force in the battle against infectious diseases, particularly its pivotal role to thwart COVID-19 (2–4, 8, 9). Recent research has shown that mRNA vaccines have therapeutic potential against solid cancers such as melanoma (10, 11), prostate (12), colorectal (13), pancreatic, head and neck cancers as well as non-small-cell lung cancer (14), and more recently, glioblastoma (15). In this review, we explore the basic components of mRNA vaccines (16), advances in mRNA vaccine design, and the potential of mRNA vaccines to treat glioblastomas, highlighting the progress made in personalized, precision mRNA medicine.

RNA technology is still in its infancy (17). Only a few years ago, almost all attention in immunotherapy was centered on the remarkable scientific and clinical advances in oncology resulting from the introduction of immune checkpoint blockade (18, 19). Although there is a distinct group of long-term survivors, including patients with metastatic cancer, most patients with cancer have recurrences and are resistant to immune checkpoint inhibitors (ICIs) when given as a single immunotherapy. Across the spectrum of human cancer, immune resistance results from an immunosuppressive, tumor microenvironment (TME) as well as insufficiency of numbers or functional, activated T cells (18). Therefore, ICIs are now being proposed to synergize within new “platforms” of cellular immunotherapy such as CAR T cells (20, 21) or dendritic cell (DC) vaccines (22).

**Figure 1:**
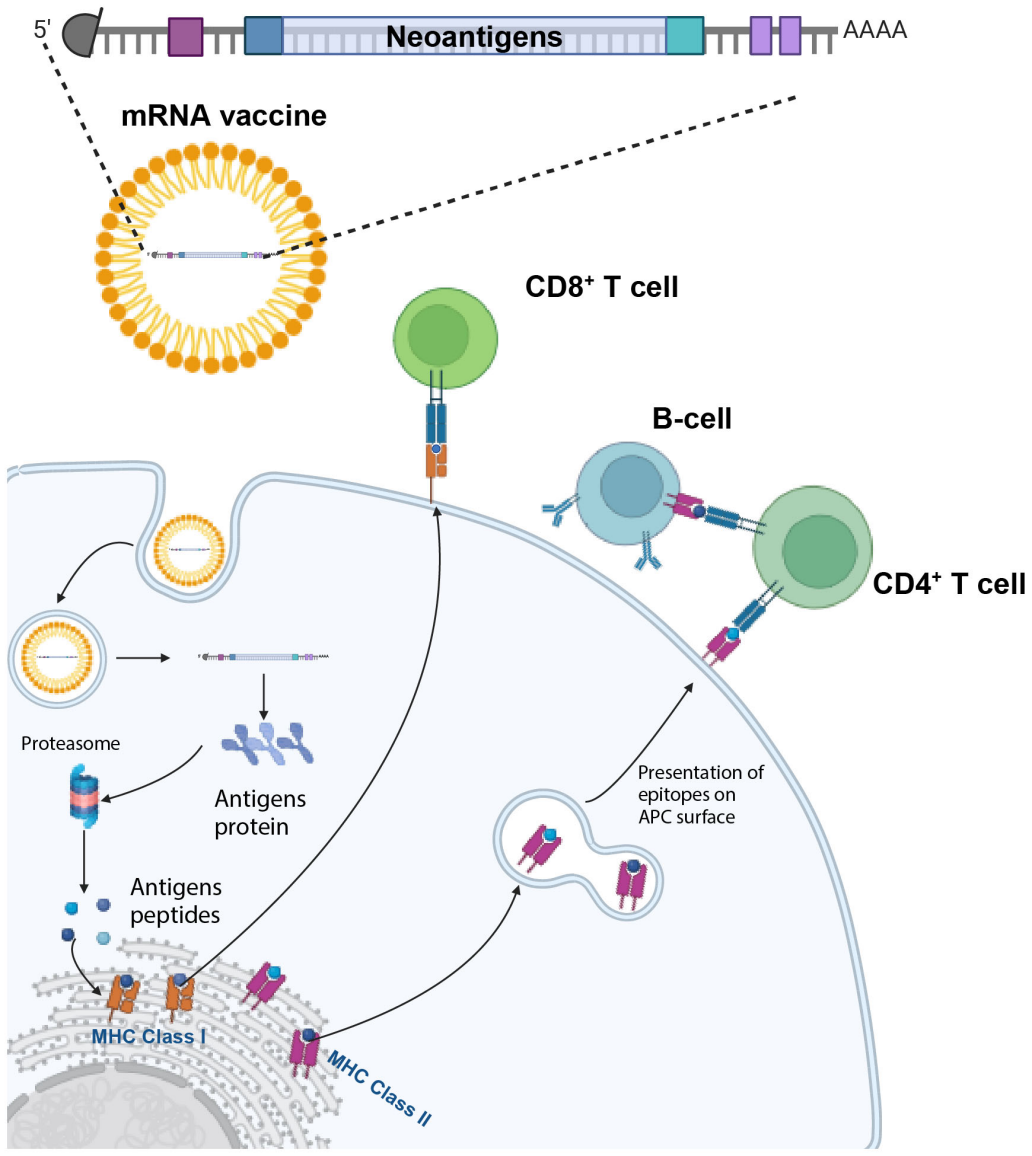
mRNA vaccines activate both humoral and cell-mediated immunity. The mRNA vaccine encoding several tumor neoantigens is injected and enters antigen-presenting cells (APCs). Here, the mRNA is endocytosed, and then translated, with the different antigens being processed by the proteasome and subsequently binds to MHC Class I molecules in the endoplasmic reticulum and are exported to the cell surface to activate CD8^+^ T cells. In parallel, the processing of antigens through the endosomal pathway enables the activation of CD4^+^ T-cells and B-cells. Created with BioRender.com.

Based on different preparation methods, platforms for cancer vaccines are divided into four categories (23): *i*) cell-based vaccines (CAR T cells, DC vaccines); *ii*) viruses-based, oncolytic vaccines (21, 24–26); *iii*) peptide-based vaccines; and *iv*) nucleic acids-based vaccines, which include DNA and RNA vaccines, composed of the encoding gene and carrier group of pathogen antigens (23). mRNA vaccines are synthesized *in vitro*, and then *in vivo* encode antigens and express proteins after internalization to stimulate an immune response (23), (**Figure 1**). In recent years, combining cancer vaccines with various immunotherapies or standard treatments has become a promising new avenue to overcome immune resistance and improve clinical outcomes (20–22).

A guide to the current concepts in the development of mRNA vaccines is featured in **Table 1**, including the comparative advantages and disadvantages of the four platforms for cancer vaccines, and their use as part of a combination regimen, as well as safety concerns (27–40). These topics will be discussed in greater detail, with an emphasis on applications to neuro-oncology (Section 4) based on the authors’ translational studies and early-stage trials for glioblastoma and in a variety of human cancers (**Table 2**).

**Table 1:** Current concepts in the development of mRNA vaccines for glioblastoma and other solid cancers: pearls and caveats in the selection, application, and combination of mRNA vaccines related to the landscape of cancer immunotherapy.

Current Concepts in the Development of mRNA Vaccines for Glioblastoma and Other Solid Cancers
1. ***Unmet Need***. Despite > 40 monoclonal antibodies and six CAR T cell therapies approved for a broad spectrum of malignancies, only a minority of cancer patients have a durable response to current immunotherapeutics (27). The only approved cancer vaccine, Sipuleucel-T (NCT00065442), an autologous dendritic cell therapy for prostate cancer, was approved in 2010, but never gained widespread use due to its high cost and underwhelming clinical efficacy (27). Currently the standard of care for glioblastoma includes surgery (maximal safe resection), chemotherapy (temozolomide) and radiation therapy with judicious use of tumor-treating fields, bevacizumab, and chemowafers. There is currently no FDA-approved immunotherapeutic regimen FDA-approved for glioblastoma. In the reignition of the Cancer Moonshot initiative by President Biden, two of the ten central research recommendations include translational immunotherapy and overcoming resistance (27).
2. ***Advantages of mRNA vaccines.*** The inherent *modularity* of mRNA-LNP vaccines enable the encapsulated mRNA to encode for *many proteins*, enables their formulation and clinical translation to be more *rapid* and *economical* than prior cell-based technologies (10, 27, 28). Specifically, manufacturing costs are low compared to other classes of vaccines (10), cost-effective and scalable – mainly due to high yields of *in vitro* transcription reactions (28). Also, there can be a “payload” targeting multiple proteins. Six of the first ten clinical trials using mRNA vaccines were individualized to a specific patient’s specific neoantigens, thus offering a more *personalized approach* than other forms of immunotherapy (27). The ability to *develop patient-specific vaccines* has the potential to elicit therapeutic responses in those recalcitrant to existing treatments (27). mRNA vaccines can induce both *humoral* and *cellular* immune responses (10). Initially, for cancer immunotherapy, mRNA was used only as a template encoding tumor-associated antigens, but due to its versatility and design variability, the therapeutic potential of mRNA is now considered limitless (10). Because patient-derived mRNA can be amplified *in vitro*, a relatively small number of cells is needed to develop a mRNA vaccine, important for patients who only have a small, surgical biopsy (29).
3. ***mRNA Vaccines and Combination Therapies.*** Beyond personalizing vaccine antigens, mRNA provides a unique opportunity to develop combination therapies. Immune stimulating mRNA into vaccine formulations can combat the immunosuppressive TME, including boosting antigen presentation and DC activation. Although not validated through testing, the current concept is that immune stimulation with mRNA could be synergistic with other vaccine types (27). A vaccine format such as a mRNA vaccine (in combination with synthetic peptides, DNA vaccine, or viral vectors) allows for targeting of dozens of mutations per patients (30). This concept of “multiple warheads,” can be used to combine complementary categories of neoepitopes such as MCH-1 and MHC-II, clonal and subclonal, undetected antigens, an approach that mitigates the risk of ‘betting on a biological hypothesis that later is proved to be wrong’ (30). Larger tumor loads, especially, might require combination immunotherapies (30). Neoepitope vaccines are safe and well-tolerated; combining them with drugs or ICIs could keep the repertoire of vaccine-induced T cell specificities functional (30). mRNA vaccines are capable of both priming and boosting immunological responses and can thus serve as an important backbone for any immunotherapeutic regimen (31)..
4. ***Safety of mRNA Vaccines.*** Vaccines that are centered on mRNA are generally considered safer than DNA and viral vectors as mRNA is the minimal genetic vector, containing only the elements directly required for the expression of the encoded protein (10). The risk of infection or insertional mutagenesis is minimal or negligible compared to viral or DNA vectors due to mRNA’s non-infectious nature and non-integration with the genome (6, 16, 23, 28, 29, 32, 33).
**5. *Comparison of advantages of the four major cancer vaccine platforms***. The pros and cons of the four major platforms/categories of cancer vaccines (23) are summarized by Fan et al. (34): • **1) Nucleic Acid-based Vaccines**: **a) DNA Vaccines:** Advantages include stability (29), low cost (35); cell-independent production; durable immune response; and potential for targeting multiple neoantigens. Once plasmid DNA enters the nucleus, a single plasmid DNA can produce multiple mRNA copies, producing more antigens than a single mRNA molecule (23). Efforts to improve immunogenicity and clinical application of DNA vaccines include electroporation, codon optimization of plasmid constructs, or co-administration of adjuvants (35). An ideal technology for cancer vaccines should allow the codelivery of multiple CD8^+^ and CD4^+^ T cell epitopes from several cancer antigens (35). The concerns include low transfection efficiency; risk of autoimmune reactions; risk of integration into host genome. b) **mRNA Vaccines**: mRNA vaccines have rapidly emerged as agents that can induce robust antitumor activity against both shared (“off-the-shelf”, mass produced, analogous to COVID-19 mRNA vaccine) and personalized antigens, with both approaches shown to be or likely to become commercially feasible in the near future (31).These are synthesized *in vitro*, encode antigens and express proteins after internalization to stimulate an immune response (23). mRNA is an ideal platform for personalized neoantigen vaccine preparation (23).Encoding full-field tumor antigens simultaneously and cross-presenting multiple epitopes of human leukocyte antigen (HLA) by APCs can induce a broader T cell response (23) Advantages, as noted, include: rapid development and easy modification; high immunogenicity; cell-independent production; able to enter non-dividing cells; intrinsic adjuvant effect; high efficiency into DCs (36). DNA molecules need to enter the cell nucleus to initiate transcription, while mRNA enters the cytoplasm to translate and express antigens directly. Therefore, mRNA antigen production is instantaneous and efficient. DNA vaccines need an extra step to go into the cell nucleus, leading to a lower immune response than mRNA vaccines (23). The concerns include fast degradation speed, especially linear mRNA (29), susceptibility to RNase degradation (37), potential for inflammatory reaction, and inefficiency of *in vivo* delivery (23) • **2) Peptide-based Vaccines**: Advantages include high specificity and safety; cell-independent production; low risk of autoimmunity; direct presentation on MHC in short peptides; proven clinical activity with synthetic long peptides. Disadvantages include high cost; complex manufacturing process; potential for HLA-restriction (32). • **3) Cell-based Vaccines:** The advantages are strong immune stimulation; multi-form antigen loading. Disadvantages include high cost; potential for undesirable immunogenicity of the cells (on target, off-tumor); and need for patient-specific customization (for autologous vaccines). • **4) Viral and Bacterial Vector Vaccines:** The benefits include high immunogenicity; long-term immune response; and self-adjuvanticity. The risks include potential for vector immunogenicity; and need for specialized storage conditions.
**6. *Comparison of mRNA vaccines with peptide vaccines.*** The early successes of mRNA vaccines could position this novel therapeutic class of vaccines as a superior “platform” compared to decades of testing with peptide vaccines that have been largely unsuccessful. mRNA vaccines provide greater flexibility, enabling the use of multiple permutations of targets, backbones, and combinations, with adaptability and encouraging progress to commercialize mRNA vaccines, making mRNA vaccines uniquely positioned to suppress malignant evolution (31), advancing the goal of “immuno-interception” (38). *Cancer is capable of progressing only when the normal function of the immune system is disrupted* (10).
**7. *Combination Therapies – Combining mRNA Vaccines with Other Vaccines and Immunomodulatory Approaches***. In recent years, combining cancer vaccines with various immunotherapies or standardized treatments has become an effective strategy for overcoming tumor resistance and improving clinical outcomes (23). Current protocols employ a multi-pronged approach that focus on three obstacles: a) T cell exhaustion with strategies to activate and refresh CD4^+^ and CD8^+^ T cells; b) the immunosuppressive TME, e.g., cytokine reprogramming using stereotactic radiation therapy, an inhibitor of IL-6 (tocilizumab) and an ICI (atezolizumab) (39); and iii) inhibition of immune checkpoint (PD-1/PD-L1) pathways using ICIs. A combination approach is also being applied to vaccine development (20, 21); For example, an mRNA vaccine, encoding a chimeric receptor directed towards CLDN6, was found to enhance the efficacy of claudin-CAR-T cells against solid tumors (40), use of a nanoparticulate RNA vaccine stimulated adoptively transferred CAR-T cells. Presentation of the CLDN6 antigen on resident APCs promoted cognate and selective expansion of CAR-T cells; improved engraftment of CAR-T cells; regression of large tumors in difficult-to- treat mouse models was achieved at subtherapeutic CAR-T cell doses (40). In the field of cancer immunotherapy, we have entered into an era of combined treatments (18, 35), and the development of potent therapeutic anticancer vaccines may be the missing element for being able to efficiently treat more patients and a wider range of tumors. There is a strong rationale for combining cancer vaccines with other immunotherapy drugs, such as immune checkpoint inhibitors or oncolytic viruses. Combining cancer vaccine and tumor resection allowed the infiltration of activated T cells to the resection site with a strong impact on mouse survival in an aggressive GBM preclinical model (35). The positive experience of combinatorial strategies for CAR T cell therapy could be extended to the future use of mRNA vaccines. For example, the use of **oncolytic viruses** leads to M1 polarization, oncolysis, damage-associated molecular patterns (DAMP)s and release of tumor antigens, resulting in enhanced T cell activation (21). Combining mRNA vaccines with CAR T cells could activate APCs, attack tumor-associated antigens leading to T cell expansion, and ultimately, cancer cell death (21). Cytokines could be added to mRNA vaccine therapy, as suggested for CAR T cell therapy (21) to reverse the immunosuppressive TME.
**8. *Combination with Immune Checkpoint Inhibitors.*** The combination of mRNA vaccines with ICIs can enhance cell-mediated immunity (10). Combined with CAR T cell therapies, ICIs enhance the function of tumor infiltrating lymphocytes (TILs), restoring their ability to attack cancer cells (21).
**9. *Results of Early Clinical Trials using mRNA Vaccines*. Although there are no FDA-approved mRNA vaccines, the results of early clinical trials are promising (** **Table 2** **), including encouraging phase II studies across various platforms, an ongoing phase III trial and auspicious data from patients with poorly immunogenic tumors** (15, 31).

**Table 2:** Active clinical trials for mRNA cancer vaccines registered on clinicaltrials.gov.

NCT Number	Study Status	Phase	Target Malignancy	Treatment- Specifics	Sponsor
NCT05192460	Recruiting	NA	Gastric Cancer, Esophageal Cancer, Liver Cancer	Neoantigen tumor vaccine +/-PD-1/L1	NeoCura
NCT05359354	Recruiting	NA	Solid Tumor	Personalized neoantigen tumor vaccine	NeoCura
NCT05981066	Recruiting	NA	Advanced Hepatocellular Carcinoma	ABOR2014/IPM511 vaccine	Peking Union Medical College Hospital
NCT03908671	Recruiting	NA	Esophageal Cancer, Non-Small Cell Lung Cancer	Personalized mRNA tumor vaccine	Stemirna Therapeutics, The First Affiliated Hospital of Zhengzhou University
NCT05940181	Recruiting	NA	Solid Tumor	Sintilimab	NeoCura
NCT05949775	Not yet recruiting	NA	Advanced Malignant Solid Tumors	Neoantigen personalized vaccine	Stemirna Therapeutics
NCT06353646	Not yet recruiting	NA	Pancreatic cancer	XH001 mRNA vaccine + Ipilimumab+ Chemotherapy	NeoCura
NCT05761717	Not yet recruiting	NA	Postoperative Hepatocellular Carcinoma	Neoantigen mRNA Personalized Cancer vaccine + Sintilimab	Shanghai Zhongshan Hospital
NCT06141369	Recruiting	NA	Adrenal Cortical, Carcinoma Medullary, Thyroid Cancer, Thymic Neuroendocrine Carcinoma, Pancreatic Neuroendocrine Tumor	Individualized mRNA neoantigen vaccine (mRNA-0523-L001)	Shanghai Jiao Tong University School of Medicine
NCT06326736	Recruiting	Early phase I	Resectable Pancreatic Cancer	Personalized vaccine SJ-Neo006 + Gemcitabine + Abraxane + Camrelizumab	Jinling Hospital, China
NCT02872025	Recruiting	Early phase I	Carcinoma, Intraductal, Noninfiltrating	Intralesional mRNA 2752 + Pembrolizumab	Merck Sharp & Dohme LLC, ModernaTX, Inc.
NCT06156267	Not yet recruiting	Early phase I	Pancreatic Cancer	mRNA tumor vaccine + Adebrelimab	Fudan University, Shanghai Regenelead Therapies Co., Ltd.
NCT05579275	Recruiting	I	Advanced Malignant Solid Tumors	Self-replicating JCXH-212 mRNA vaccine	Peking University Cancer Hospital & Institute
NCT05738447	Recruiting	I	Liver Cancer, Hepatocellular Carcinoma	HBV mRNA vaccine	West China Hospital
NCT06019702	Recruiting	I	Digestive System Neoplasms	Ineo-Vac-R01	Sir Run Run Shaw Hospital, Hangzhou Neoantigen Therapeutics Co., Ltd.
NCT05198752	Recruiting	I	Solid Tumor	Personalized neoantigen mRNA cancer vaccine	Stemirna Therapeutics
NCT06026800	Recruiting	I	Digestive System Neoplasms	Ineo-Vac-R01 + standard first line treatment	Sir Run Run Shaw Hospital, Hangzhou Neoantigen Therapeutics Co., Ltd.
NCT04745403	Recruiting	I	Hepatocellular Carcinoma	MRNA HBV/TCR T-cells	Lion TCR Pte. Ltd.
NCT05938387	Active Not Recruiting	I	Glioblastoma	CV09050101 mRNA vaccine	CureVac
NCT05714748	Recruiting	I	Malignant Tumors	EBV mRNA vaccine	West China Hospital
NCT06026774	Recruiting	I	Digestive System Neoplasms	Ineo-Vac-R01 + standard adjuvant therapy	Sir Run Run Shaw Hospital, Hangzhou Neoantigen Therapeutics Co., Ltd.
NCT05264974	Recruiting	I	Melanoma	Autologous total tumor mRNA loaded DOTAP liposome vaccine	University of Florida
NCT04573140	Recruiting	I	Adult Glioblastoma	Autologous total tumor mRNA and pp65 LAMP mRNA loaded DOTAP liposome vaccine, RNA-LPs	University of Florida, Pacific Pediatric Neuro-Oncology Consortium, University of California, San Francisco, CureSearch, Team Jack Foundation, Florida Department of Health
NCT05942378	Not yet recruiting	I	Advanced Solid Tumor	HRXG-K-1939 mRNA vaccine + Adebrelimab	Fudan University
NCT06195384	Not yet recruiting	I	Solid Tumor, Adult	Neoantigen mRNA Vaccine	Second Affiliated Hospital of Guangzhou Medical University
NCT05978102	Recruiting	I|II	Advanced Solid Tumor	STI-7349 mRNA + Pembrolizumab	The Fourth Affiliated Hospital of Zhejiang University School of Medicine
NCT06273553	Not yet recruiting	I|II	HPV- Associated Intraepithelial Neoplasia	RG002 mRNA vaccine	RinuaGene Biotechnology Co., Ltd.
NCT06249048	Not yet recruiting	I|II	Advanced Solid Tumor	STX-001 mRNA vaccine+ pembrolizumab	Strand Therapeutics Inc.
NCT04534205	Recruiting	II	Unresectable, Metastatic or Recurrent Head and Neck Squamous Cell Carcinoma	Bnt113+ pembrolizumab	BioNTech SE
NCT03688178	Active Not Recruiting	II	Glioblastoma	Human CMV pp65-LAMP mRNA-pulsed autologous DCs + Temozolomide; Varlilumab, Unpulsed DCs	Celldex Therapeutics
NCT03897881	Recruiting	II	Melanoma	MRNA-4157+Pembrolizumab	ModernaTX, Inc.
NCT03815058	Active Not Recruiting	II	Untreated Melanoma	Autogene cevumeran+ Pembrolizumab	Genentech, Inc.

NA, not applicable.

An important, but nuanced, biological advantage of mRNA vaccines is the recent discovery that in order for immunotherapy to eliminate solid tumors, there needs to be a functioning intratumoral “triad” of synergistic activity between *i*) antigen-presenting cells (APCs)/dendritic cells; *ii*) activated CD4^+^ T cells and *iii*) activated CD8^+^ T cells which licenses CD8^+^ T cell cytotoxicity and elimination of cancer cells (41). mRNA vaccines are in a unique position to activate each of these three, critically important cell subpopulations by the method of uptake in the APC and the activation of both CD8^+^ cells CD4^+^ T cells through binding on the cell surface, respectively, to MHC (major histocompatibility class) I and II molecules (**Figure 1**). and then activation of the T cell receptor (28).

## 2 Mechanism of mRNA vaccine-mediated activation of anti-tumor immunity

Broadly speaking, mRNA cancer vaccines consist of mRNA molecules encoding specific tumor antigens. Upon administration, these mRNA molecules are subsequently internalized by APCs where they undergo translation, resulting in the production of protein antigens. These antigens are further processed into antigen peptides, which subsequently bind to MHC 1 molecules within the endoplasmic reticulum and are then presented or cross-presented on the surface of APCs (42, 43). This process activates CD4 ^+^ and CD8^+^ T cells, orchestrating a potent cell-mediated immune response (**Figure 1**). In parallel, protein antigens encoding mutated peptides are routed through the endosomal pathway. This alternative route enables the activation of CD4^+^ and CD8^+^ T cells through MHC Class I/II presentation (44). This dual activation of both CD8^+^ and CD4^+^ T cells amplify the breadth and potency of the immune response. Dual activation of both CD8^+^ and CD4^+^ T cells as well as APCs are required to successfully eliminate solid tumors, otherwise refractory to immunotherapy (41).

What are the specific steps by which targeted mRNA is internalized by the APC to trigger an immune response by releasing the translated antigen or presenting the epitopes onto the surface of cells? One model (28) describes a sequence of nine steps*: i)* the targeted mRNA-LNP binds to the cell surface receptor of the APC mediated by specific ligands; receptor activation can lead to interferons or other cytokine/chemokine production; *ii)* after endocytosis, mRNA in the endosome interacts with membrane-bound Toll-like receptors (TLRs); *iii)* triggering of TLR activates signal transduction pathways that selectively lead to production of Type 1 interferons (45) that upregulate the effector function of immune cells (e.g., DCs, T cells, and B cells) and/or pro-inflammatory cytokines; *iv)* entrapped mRNA then is released from the endosome into the cytosol where *v)* the mRNA is translated by ribosomes; *vi)* upon translation, the proteins are either a) secreted out of the host APC, or b) processed within the APC by the proteasome into smaller antigen peptides; *vii)* secreted extracellular mRNA is then taken up by another APC, degraded into peptides; these epitopes are subsequently presented on the cell surface by MHC class II molecules*; viii);*alternatively the intracellular peptides are processed within the endoplasmic reticulum and loaded onto MHC class I and/or class II molecules *ix)* the epitopes bound to MHC class I/II molecules migrate to the cell surface where they bind to the T cell receptor (TCR) of CD8^+^ and/or CD4^+^ T lymphocytes (28).

Furthermore, the secreted protein antigen, encoded by the mRNA vaccine, plays a critical role in stimulating B cells. This activation prompts the production of neutralizing antibodies, thereby bolstering the humoral arm of the immune response. In summation, mRNA vaccines exhibit remarkable potential in eliciting a comprehensive immune response against tumors by instigating both robust humoral and cell-mediated immunity (**Figure 1**). Four pivotal aspects come into play in the creation of an effective mRNA cancer vaccine: *i)* identification of tumor antigens; *ii*) vector design; *iii)* delivery; and *iv*) manufacturing.

### 2.1 Identification of tumor antigens

The accumulation of genetic mutations in cancer leads to the creation of unique tumor-specific antigens or neoantigens (46). These unique antigens can be displayed by the major histocompatibility molecules found on the surface of tumor cells. T-cells primed to identify these neoantigens launch targeted assaults on cancerous cells expressing these mutations (47). In the pursuit of neoantigens, most studies have concentrated on indels and non-synonymous single nucleotide variants (SNVs). Yet, numerous SNVs are unique to individual patients, and tumors with low mutational burden exhibit a small number of SNVs that is inadequate for vaccine design (48, 49). As a result, exploring supplementary reservoirs of cancer neoantigens, like gene fusions, alternative splicing variants, and post-translational modifications, holds promise in unearthing fresh targets for immunotherapeutic interventions (50).

### 2.2 mRNA vector design

**Figure 2:**
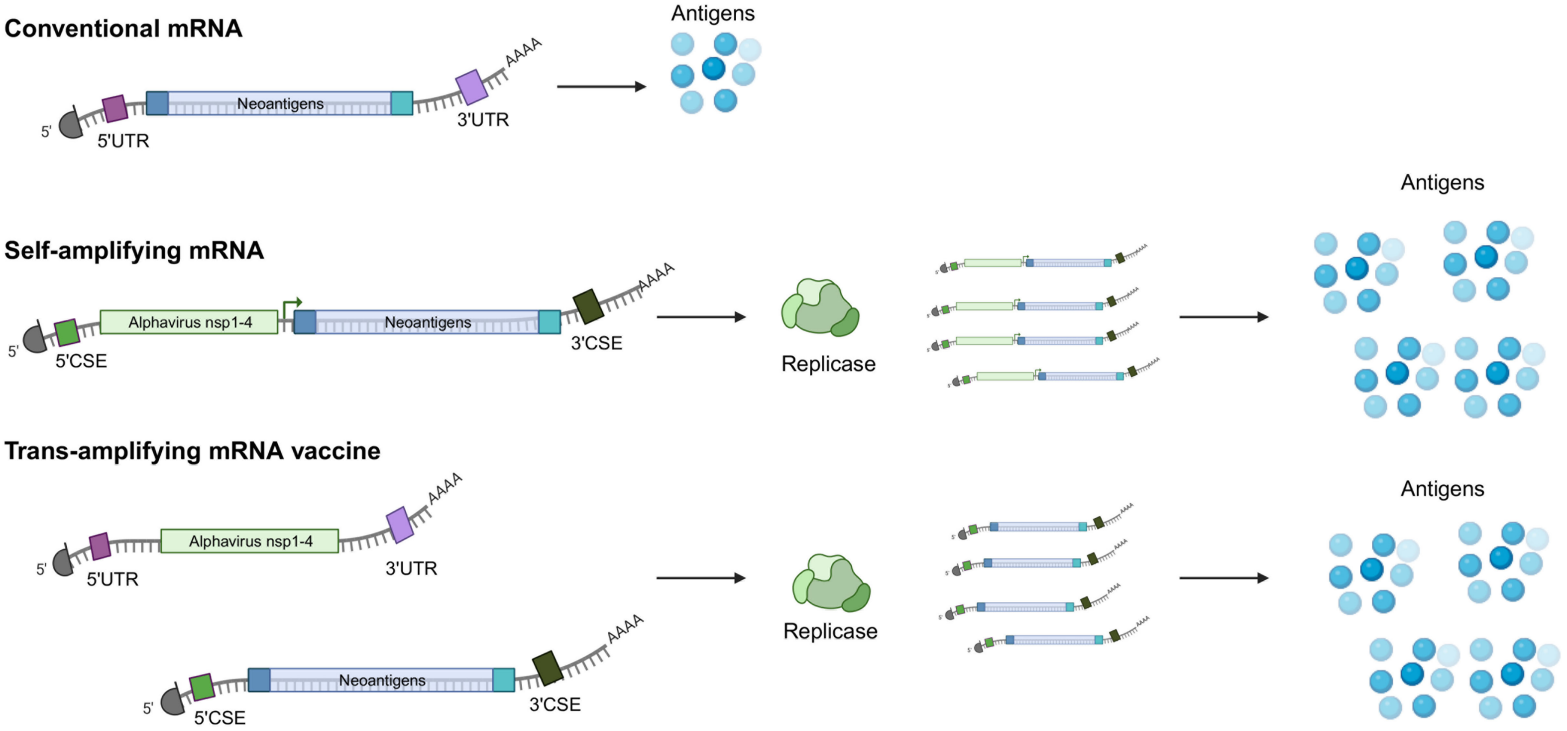
Conventional and self-amplifying mRNAs. (top) Conventional mRNAs contain the nucleoside-modified coding elements targeting tumor antigens, 5’ and 3’ untranslated regions, a polyA tail as well and a 5’ cap analog, which have all been designed to improve stability and translational potential. (middle) Self-amplifying mRNAs also encode an RNA polymerase, usually derived from alphaviruses as well as a 5’ and a 3’ conserved sequence element (CSE). The viral RNA replicase recognizes the structural CSE elements and directs the synthesis of negative-sense RNA intermediates, which are transcribed into many copies of the coding mRNA template and amplified antigen expression. (bottom) Another strategy of self-applying mRNA vaccine involves using two distinct mRNAs, one encoding for the replicase and one for the tumor antigens. Both types of self-amplifying strategies result in enhanced expression and prolonged expression of the encoded tumor antigen. Created with BioRender.com.

In terms of mRNA vector design, several strategies are employed. The conventional mRNA encodes the vaccine immunogen, flanked by 5′ and 3′ UTRs, along with a 5’ cap and polyA tail optimized for maximum stability and translational potential. In addition, many of the licensed SARS-CoV-2 vaccines contain nucleoside-modified mRNAs, using an N1-methylpseudouridine, which counters immune-related inhibition of translation and degradation (1, 51). This configuration allows for the translation of the antigen from the nonreplicating transcript (52). One drawback of conventional mRNA vaccines is the limited antigen expression, which is proportional to the number of mRNA transcripts that are delivered, thus necessitating larger doses of vaccine or repeat administrations. One way to overcome this limitation is the use of self-amplifying mRNAs. This alternative strategy employing self-amplifying mRNA has additional elements such as 5′ and 3′ conserved sequence elements (CSE), the nsP1-4 genes, and a subgenomic promoter of an alphavirus, and the vaccine immunogen (52, 53). Post-*in situ* translation, both the antigen and RNA-dependent RNA polymerase are generated (**Figure 2**). The latter identifies the CSEs, subsequently amplifying the vaccine-encoding transcripts, resulting in an augmented accumulation of tumor antigens within the cell (**Figure 2**). Trans-amplifying mRNAs introduce two distinct transcripts into the equation. One encodes for the RNA-dependent RNA polymerase (nsp1-4), while the other encodes the CSE and the viral antigen. This dual-transcript configuration achieves an even stronger self-amplifying effect (52) (**Figure 2**).

## 3 Delivery systems for mRNA vaccines

Various delivery systems facilitate the deployment of mRNA vaccines. These encompass lipid-based, polymer-based, and emulsion-based delivery systems, all utilizing cationic molecules to transport the anionic mRNA across the cell membrane (53). Critical elements of the mRNA delivery system include achieving optimal intracellular and targeted delivery, ensuring stability to facilitate antigen translation, and triggering appropriate immune activation (54).

To this end, the lipid nanoparticle (LNP) system has been recognized as a powerful and versatile delivery platform (55). These LNPs have an ionizable lipid, a helper lipid, cholesterol, and a PEG-conjugated lipid (54). A crucial aspect of the LNP system lies in its utilization of pH-sensitive cationic lipids, which facilitate cellular internalization via receptor-mediated endocytosis. The low pH within the endosome causes the ionization of cationic lipids, which interact with anionic lipids on the endosomal membrane, leading to the disruption of the endosomal membrane and release of the mRNA into the cytoplasm (53). The helper lipid, usually a phospholipid, helps stabilize the LNP structure, the cholesterol promotes membrane fusion and prolongs the half-life, while the PEG-conjugated lipid increases particle stability (56). Advances in high throughput screens and rational design approaches have yielded specific ionizable lipids tailored for diverse applications, such as systemic delivery for the SARS-CoV-2 vaccine (57) and targeted delivery to the lung epithelium (58) or placenta (59) for CRISPR-editing purposes. To identify mRNA delivery vehicles that facilitate mRNA delivery *in vivo* and provide potent, specific immune activation, a heterocyclic lipid formulation was found to demonstrate robust immune responses and tumor growth inhibition in melanoma and human papillomavirus E7 tumor models via the STING pathway, with minimized systemic cytokine expression (60).

Additional novel mRNA-LNP delivery approaches include devising targeting approaches to specifically deliver the mRNA payload into cell types once deemed inaccessible (61). Passive targeting approaches require intratumoral administration, however, the injected particles are heterogeneously distributed throughout the tumor and often accumulate in the liver and lymphatic organs (62). Additional active strategies require modifying the surface of mRNA-LNPs to allow for delivery to specific cells, for example by functionalizing antibodies on LNPs or including tRNAs with cell-type expression patterns in the cargo (61). Recently, a novel platform of activated LNPs with surface-conjugated human CD3 and CD28 antibody fragments has been introduced as a rapid, one-step method to enhance mRNA CAR T cell therapy to decrease tumor burden, and the potential to reduce the complexity, cost and time of mRNA CAR T cell production as well as to support other immunotherapy applications (63). Targeting brain cancers represents a particular challenge because of the blood-brain barrier; recently, a specific class of LNPs with structurally diverse ionizable lipids shows promise to traverse the blood-brain barrier (64).

Advances continue to be made to all individual elements of mRNA vaccine from novel types of tumor antigens and self-amplifying mRNA vectors to targetable LNPs. Focusing on several difficult-to-treat cancers, this review describes recent advances in mRNA vaccines for solid tumors outside of the CNS, such as pancreatic cancer, head and neck cancers, melanoma, and then focuses on the challenge of glioblastoma.

## 4 mRNA vaccines in human cancer

### 4.1 Pancreatic cancer

The transformative potential of mRNA vaccines is best demonstrated by recent breakthroughs in one of the most formidable cancers, pancreatic carcinoma (65, 66). Pancreatic cancer has one of the highest death rates of any solid organ malignancy, with an overall 5-year survival of less than 10%; it is currently the third most common and on a projected course to become the second most common cause of cancer-related deaths in the United States by 2030 (67–69). Surgery currently is the only modality that offers a chance of a cure (67), but 5-year survival rates after surgical resection alone are low, approximately 10% (67, 70), and up to 30% with resection and adjuvant chemotherapy (68, 70). Unfortunately, only 10-20% of patients are diagnosed with localized, surgically resectable disease (68), and over 90% relapse 7-9 months after resection (70). Pancreatic cancers have historically shown resistance to immunotherapy, partly attributed to a complex immunosuppressive microenvironment, poor T cell infiltration, and reduced mutational burden leading to reduced activation of antitumor T cells (71, 72). In addition, pancreatic cancer is thought to harbor very few neoantigens (an average of 35 compared to hundreds in melanoma), thus having weak antigenicity (71, 73, 74). Multiple pancreatic cancer immune subtypes have been identified. For example, pancreatic tumors categorized as immunologically “cold” typically exhibit low immunogenicity and/or a high presence of reactive stroma (75). Pancreatic adenocarcinoma, akin to glioblastoma, has proven almost entirely insensitive to immune checkpoint inhibition with a response rate < 5% (70); this insensitivity can be partially ascribed to the low mutation rate, and the consequent scarcity of neoantigens (70) as well as intratumoral and inter-tumoral heterogeneity (76). Thus, a combination of a personalized mRNA vaccine with immunogenic chemotherapy, stromal modulation, and ICI may be needed for an effective therapy (76).

Despite these challenges, Rojas and colleagues conducted a phase I clinical trial that implemented a personalized mRNA vaccine strategy, wherein at least five, and up to 20 neoantigens specific to each patient’s tumor, were identified and integrated into the vaccine (65). The vaccine was delivered using lipoplex nanoparticles via intravenous injection after surgical resection and in combination with the standard mFOLFIRINOX chemotherapy. Notably, all participants also received a single dose of an ICI before receiving their personalized mRNA vaccine. Encouragingly, T cells recognizing specific neoantigens were detected in half of the trial participants, categorized as immune responders. Strikingly, immune responders showed no signs of cancer recurrence at a median follow-up of 18 months, compared to a median time to recurrence of 13.4 months in non-responders (65). Nevertheless, treating pancreatic cancer remains challenging as half of the participants did not respond to the vaccine and most patients were not eligible for surgery and thus ineligible for the vaccine. Strategies to boost the response to the vaccine and predict responsiveness will be an advance to enrich the percentage of responders. One possibility, going forward, would be to treat patients harboring unresectable cancers with FOLFIRINOX neoadjuvant chemotherapy who then might qualify for the surgery and enable them to get the personalized mRNA vaccine (77, 78).

### 4.2 Head and neck cancers

Head and neck squamous cell carcinomas (HNSCCs), arising from the mucosal epithelium, represent the most prevalent histological type of head and neck malignancy (79). These cancers are characterized by their multifactorial etiology, stemming from infections with high-risk human papillomaviruses (HPVs) (80–82) or Epstein–Barr virus (83, 84) and lifestyle-related risk factors including alcohol consumption and smoking (85, 86). Despite significant advancements in treatment modalities for HNSCCs, encompassing surgical interventions, radiotherapy, and chemotherapy, the 5-year overall survival rate remains in the range of 40–50%; however, the use of ICIs (e.g., pembrolizumab or nivolumab) has led to superior outcomes, leading to the integration of immunotherapy for this challenging disease (87). However, based on clinical trials (88–90), less than a third of patients respond to immunotherapy (91); therefore, additional therapies such as mRNA vaccines are needed. Given the diverse etiologies of HNSCC, the HNSCC-associated neoantigens can broadly be divided into either virus-derived tumor antigens or non-virus-derived. HNSCC arising due to persistent infection with high-risk human papillomavirus 16 (HPV-16) is associated with improved survival (92, 93), likely due to the enhanced immunogenicity of HPV-derived neoantigens.

The potential of mRNA vaccines for HPV-specific-HNSCC has also been recently shown in murine models. The most common HPV subtype found in HPV-positive HNSCC is HPV-16, which accounts for over 90% of HPV-positive HNSCC (94). While the majority of HPV infections are cleared, infections in the epithelium of palatine and lingual tonsil can persist (95), leading to constitutive expression of E6 and E7 oncoproteins (96). Mouse model experiments with mRNA vaccines against E7 promoted tumor regression, prevented relapse, and re-sensitized mice to PD-L1 immunotherapy, rendering anti-PD-L1 refractory tumors responsive (96). Similarly, mouse model experiments using three different mRNA platforms, an unaltered non-replicating mRNA vaccine, a modified non-replicating mRNA vaccine with modified nucleosides, and a self-amplifying mRNA vaccine, showed that a single injection led to significant control of tumor growth in two murine models of HPV-16 tumors (97). From the foundation provided by these studies, current clinical trials are underway. For example, a phase II clinical trial (AHEAD-MERIT) using the BNT113 mRNA, encoding HPV16-derived neoantigens E6/7 is administered with and without pembrolizumab to treat HPV16-positive HNSCC expressing the PD-L1 protein (NCT04534205) (**Table 2**).

Several studies are also emerging to assess the potential of mRNA vaccines against non-viral HNSCC neoantigens. Chen et al. used The Cancer Genome Atlas (TCGA) and the Gene Expression Omnibus databases to analyze alternative splicing and mutations of genes with HNSCC (98). Seven potential tumor antigens, [SREBF1, LUC7L3, LAMA5, PCGF3, HNRNPH1, KLC4, and OFD1], which were associated with nonsense-mediated mRNA decay factor expression, overall survival, and infiltration of APCs and would thus induce a potent anti-tumor T-cell response. Furthermore, the authors used clustering analysis to select suitable patients whose immune subtypes made them likely to respond to vaccination. Potential biomarkers included several genes that were identified to serve as potential prognostic biomarkers for mRNA vaccines: IGKC, IGHV3-15, IGLV1-40, IGLV1-51, IGLC3, IGLC2, and CD79A (98). To further distinguish the immune subtypes of HNSCCC to select suitable patients for vaccination, another group identified three genes as targets for developing mRNA vaccines: CCR4, TMCO1, and SPACA4 that were upregulated, and correlated with survival and tumor infiltration by both B and T cells, inducing a potent immune response (99). Paradoxically, patients with immune subtype C3, or the immune “cold” subtype – tumors with a lower IFN-γ and TGF-β response, fewer macrophages, T cells, and CD4 memory responses–were most likely to respond to mRNA vaccines against HNSCC (99). The authors speculate that mRNA vaccines would be most effective in transforming tumors that have a “cold” (immunoresistant) TME (100). Recognizing that histologically distinct tumors have unique immune landscapes, if these results apply to glioblastomas, it would further support the use of mRNA vaccines for human glioblastoma, a tumor known to be characterized by a “cold” TME.

### 4.3 mRNA vaccines in other non-CNS human cancers

Two clinical trials with personalized mRNA vaccine encoding neoantigens are underway in China, for patients with advanced esophageal cancer and non-small cell lung cancer (NCT03908671), and advanced gastrointestinal cancer (esophageal, liver, and advanced gastric cancer (NCT05192460). Additional trials are underway and include mRNA vaccines designed for patients with liver cancer (NCT05761717), and endocrine cancer (NCT06141369). Trials are also underway for bladder cancer (100), melanoma, prostate cancer, breast cancer, and other solid tumors as detailed in **Table 1**. In patients with stage IIB to stage IV resected melanoma, ICIs are standard therapy, but many patients recur; when a mRNA-vaccine, individualized therapy (mRNA-4157) is added to ICI (pembrolizumab),18-month recurrence-free survival was increased in the combination group (79%) compared to ICI alone (62%) with a hazard ratio for recurrence or death of 0.53, p=0.05 (NCT03897881, KEYNOTE-942) (11). Importantly, there was a lower recurrence or death rate (22%) in the combination group compared to 40% in the group treated with ICI alone (11). A phase I trial of intratumoral STX-001, a novel LNP, self-replicating mRNA expressing the cytokine IL-12 for an extended duration, is being evaluated in advanced, treatment-refractory solid tumors (NCT06249048) (101).

## 5 Brain tumors: pediatric and adult gliomas

Novel approaches to glioblastoma are urgently needed because standard therapy is associated with a median survival of eight months, and a five-year survival of 6.9% (102). Numerous biological barriers to immunotherapy include cellular heterogeneity, plasticity, and an immunosuppressive TME (103, 104). Immune cells constitute an important component of the glioma microenvironment, constituting as much as 50% of the tumor mass (103). Glioblastoma is immunologically “cold” with a TME resistant to T-cell and DC infiltration (105). Furthermore, there is *i*) a scarcity of circulating T cells with sequestration of T cells in the bone marrow; *ii*) a localized immunosuppression due to secretion of immunosuppressive cytokines, such as TGF-β, IL-6, PGE_2_ and *iii*) an upregulation of PD-1 and PD-L1 (106, 107). Compared to tumors such as melanoma with a high mutation burden, glioblastoma has a reduced array of immunogenic neoantigens (107). Despite these challenges, several recent studies have sought to broaden the pool of targetable neoantigens in glioblastoma, offering potential avenues for mRNA vaccines (108–110). Given the substantial challenges, there is recent, encouraging data showing biological and clinical evidence of converting the glioblastoma TME into an immune responsive environment (15, 24, 111–117). It appears that a multimodal approach using an mRNA vaccine in combination with other strategies to boost the immune system could ultimately extend survival and change the outlook for patients. Recent advances in six pillars of immunotherapy are summarized:

**a) mRNA vaccine**. In a first-in-human clinical trial (NCT04573140, **Table 2**), Mendez-Gomez et al. recently reported a striking expansion of the immune response to tumor-associated antigens in patients with glioblastoma, using a novel RNA lipid particle aggregate (LPA), associated with a clinical increase in overall survival (15). The LPA differs from the commonly used LNPs (**Figure 1**) that are size-limited to permit endocytosis; by contrast, the LPA-based mRNA vaccine does not rely on TLR for engagement, enabling the delivery of multiple mRNA payloads to the same cancer cell, as shown in a canine model of glioma using the LPAs to elicit a potent RIG-I (retinoic acid-inducible gene I protein)-mediated stimulation of the immune system (15). Additional candidate genes are being identified by mining databases, including the TCGA and the Chinese Glioma Gene Atlas to identify multiple genes suitable for mRNA vaccine development (108, 110, 118).

**b) DNA vaccine.** Advantages of DNA vaccines include stability, relatively low-cost, cell-independent production, a durable immune response, and potential for targeting multiple neoantigens (**Table 1**). *hTERT* (human telomerase reverse transcriptase), regarded as the first truly universal tumor antigen (119), a surprisingly immunogenic target that is fundamental to oncogenesis (20). Vaccination with *hTERT* DNA is being used for “immuno-interception” in individuals with *BRCA1* or *BRCA2* mutations and therefore at high risk of breast, ovarian, pancreas, prostate, and other cancers (NCT04367675) (120). Using a similar DNA vaccine (NCT03491683), given by electroporation, targeting *hTERT* (INO-5401) combined with an IL-12 DNA plasmid (INO-9012) and a PD-1 inhibitor (cemiplimab), Reardon et al. reported promising survival results for patients with glioblastoma with activated CD4^+^ and CD8^+^ T cells (121). The tumor tissue, post-treatment, showed genomic alterations linked to activation of the immune system, and evidence of T cell infiltration and cytolysis (121). A new generation of DNA vaccines with plasmids encoding T cell tumor epitopes (pTOP) significantly increased survival in preclinical models (GL261) of glioblastoma (35). Interestingly, vaccine monotherapy by itself was ineffective, but surgical resection of glioblastoma, followed by the vaccine, resulted in a dramatic increase in survival and delayed recurrence, associated with infiltration of activated T cells to the resection site (35).

**c) Dendritic cell vaccine**. Because APCs, such as dendritic cells, are key to initiating antigen-specific immune responses (41), early work to develop immunotherapy for cancer involved DC-mRNA vaccines (16, 122). A review of 33 early clinical trials revealed the potential of DC vaccines for glioblastoma, “*we can expect immune modulation to make its way into standard therapeutic protocols in neuro-oncology … in the near future, surgery, cytotoxic therapies (i.e., radio-chemotherapy), and immunotherapy will form a three-pronged therapeutic approach that will enhance clinical outcomes* (123). Indeed, a significant survival benefit was reported for patients with newly diagnosed (112) and recurrent glioblastoma (112, 124), with meaningful “tails” in the Kaplan-Meier survival curves, reflecting long-term survivorship. Furthermore, adding additional agents such as pembrolizumab (125) or poly-ICLC (111) can further activate the immune system, detected by a polarized interferon response in circulating monocytes and CD8^+^ T cells, translating to prolonged survival and delayed disease progression in the responders (111). RNA-pulsed DCs, using nanoparticles, are safe and under evaluation (NCT04573140) (32).

**d) CAR T cell therapy**. mRNA vaccines show potential in combination with CAR T cell approaches to treat intractable pediatric brain tumors (126). The mRNA vector is expressed only transiently, which minimizes off-target toxicity, especially in the brain (127). The use of mRNA-CAR constructs prolonged survivals in preclinical models of diffuse midline glioma and medulloblastoma targeting GPC2 (127). Clinical trials are underway to target GPC2 in patients with neuroblastoma (NCT05650749). CAR T cell therapy is also being evaluated in pediatric high-grade gliomas targeting B7-H3 HER-2 (NCT03500991), and GD2.C7R (NCT04099797) (128). For adult human glioblastoma, clinical studies have shown that CAR-T cells can feasibly traffic to active regions of glioblastoma with on-target, biological activity (129, 130). Recent advances in patients with recurrent glioblastoma show that intrathecal delivery of CAR T cells targeting IL13α2 (NCT002208362) (116), or bivalent CAR T cells targeting two antigens, EGFR and IL13α2, (NCT05168423) (113), and EGFR/EGFRvIII with a T-cell engaging antibody, TEAM, (NCT05660369) (115), leads to compelling results (117) assessed by CAR T cell proliferation, rapid reduction in tumor size, bioactivity and safety signals. The next challenge is to transform the transient responses into long-term outcomes, converting an otherwise fatal glioblastoma into a chronic, treatable disease (117). The use of mRNA-targeted CAR T cells (131, 132) or the use of CAR natural killer cells instead of T cells (128), could be additional steps to provide durable responses. The fourth generation of CAR *T cells redirected for universal cytokine-mediated killing* (TRUCKs) results in simultaneous CAR T-Cell mediated killing and immune modulation of the TME via secretion of cytokines that has the dual effect of enhancing the survival of CAR T cells and modulating the TME by repolarizing tumor-associated macrophages or activating natural killer cells (133). Multiple phase 1 trials (NCT03542799, NCT03932565) use TRUCKS for systemic cancer (133), opening the potential combination of TRUCKs with a personalized mRNA vaccine.

**e) Viral oncolytic therapy**. One of the main immunotherapeutic platforms consists of viral oncolytic therapy (23–26), which has the dual effect of *i*) direct killing of tumor (glioblastoma) cells and ii) the dying cells release neoantigens that can attract APCs and, in turn, activate CD4^+^ and CD8^+^ T cells. Many viruses have been re-engineered as vectors for gene therapy of glioblastoma, e.g., retroviruses, adenoviruses, or herpes-simplex type 1 viruses (134). Other viruses have been engineered to replicate within brain tumors in a limited manner without causing encephalitis. To increase the effectiveness of oncolytic herpes virus, Todo et al. injected active virus into the surgical resection cavity, or unresectable tumor, up to six times (135). An alternative, novel, minimally invasive approach to treat glioblastoma is to develop viral vectors using variants of the capsid of adenovirus, AAV9, that bind to the transferrin receptor BI-hTFR1, allowing efficient transfer of genes across the blood-brain barrier, and delivered via the systemic circulation rather than direct injection (136).

The use of mRNA vaccines that leverage the genome of oncolytic viruses holds great promise to treat glioblastoma (137). Studies aimed at identifying potential antigens in glioblastoma (GBM) for the development of advanced mRNA-based therapies identified numerous distinct antigen sets, thereby meeting the challenge of comprehensive, multimodal treatment (137, 138). Initial results of ABTC 1603 (NCT00589875), using an adenovirus-tk (CAN-2409) in combination with an ICI (nivolumab), are promising, suggesting a survival advantage (139). A first-in-human trial of CAN-31100, an engineered herpes simplex 1 virus, shows safety signals and may extend survival by immune stimulation-especially in patients with antibodies to HSV1 (26). As proof of concept that oncolytic viruses can overcome the immunosuppressive TME, a combination of reovirus and CAR T-cells caused the expansion of T cells and cured > 80% of mice with intracranial EGFRvIII tumors (140). In a phase I-II trial, the use of intratumoral, oncolytic DNX-2401 virotherapy, followed by pembrolizumab, was well-tolerated in patients with recurrent glioblastoma, with notable survival benefit in select patients (141). Specifically, objective responses led to longer survival; 56.2% of patients had a clinical benefit, defined as stable disease or objective response (141). In a separate study, patients with recurrent glioblastoma, injected with an oncolytic herpes virus showed improved survival in individuals seropositive for HSV1, associated with immunoactivation – changes in the tumor/PBMC T cell counts, peripheral expansion of specific T cell clonotypes, and tumor transcriptomic signatures of immune activation (26). These results provide validation in patients that intralesional oncolytic HSV treatment enhances anticancer immune responses, even in the immunosuppressive TME, especially in individuals with cognate serology to the injected virus (26).

**f) Cytokine reprogramming of the glioblastoma microenvironment.** In preclinical models, targeting IL-6 leads to a remarkable change in the TME, with a “switch” from the M2 immunosuppressive, (pro-tumorigenic) macrophage phenotype to an immunostimulatory (M1) phenotype, resulting in a significant increase in survival (142). Adding CD40 agonist enhanced the activity of infiltrated T cells, and an almost complete cure in glioblastoma models (143). Adding immune checkpoint inhibitors further improves survival (143, 144). Taken together, these findings led to an ongoing multicenter trial, NRG-BN-010 (NCT047299959), combining inhibition of IL-6R (tocilizumab), PD-L1 (atezolizumab) and stereotactic radiosurgery to treat recurrent glioblastoma (39). Recently, IL-6 blockade was found to promote tumor immunity through activation of the immunostimulatory IL-12 pathway, while abrogating the toxicity of checkpoint blockade, thus decoupling tumor immunity from autoimmune toxicity (145). Taken together, combining anti-IL6 blockade with a mRNA vaccine would be an attractive approach. One caveat, however, is the LNPs that coat the mRNA are by themselves immunostimulatory, acting as an adjuvant component, fostering T-follicular helper cells (Tfh cells) and humoral responses that are abrogated if Il-6 induction by the LNP is blocked using an antibody or using Il-6 deficient mice (55); the implications for cancer therapy in humans are unknown. Another approach to cytokine reprogramming is the use of convection-enhanced delivery and targeting of the IL-4 signaling pathway (NCT02858895), producing a dose-dependent, survival benefit with a high-dose immunotoxin (bizaxofusp) that targets the interleukin-4 receptor, IL4R (146). Single treatment with bizaxofusp increased median overall survival by up to 50% and 12-month progression-free survival by almost 100% when compared to FDA-approved therapies (146). A novel method to convert the immunosuppressive TME of glioblastoma is to arm CAR T cells with a dominant-negative TGF-β receptor II which in a rodent model of glioblastoma lowers the levels of the immunosuppressive cytokine TGF-β in the TME, enhances T cell proliferation, eradicates intracranial tumors, and significantly improves survival (114).

**g) Immune checkpoint inhibitors in combination with mRNA vaccine.** A synergistic effect of mRNA vaccines with ICIs is reported in glioma models, with a favorable shift in the TME from an immunologically “cold” resistant environment to one that is “hot,” associated with improved survival (110, 147). Multimodal immunotherapy with ICIs for glioblastoma is under active investigation (39, 148, 149) and has been effective in preclinical models (143). Ultimately, there is a large body of evidence that a mRNA vaccine for human glioblastoma would benefit from the use of concomitant ICIs.

## 6 Challenges and caveats

In addition to the identification of the optimal tumor antigens to target in glioblastoma, key issues include delivery systems that can traverse the blood-brain barrier as well as boosting antigen production. An entirely novel method to meet this challenge is to harness the power of machine learning to reprogram glioblastoma cells into APCs that function like dendritic cells in terms of phagocytosis, direct presentation of endogenous antigens, cross presentation of exogenous antigens, and priming of naïve CD8^+^ cytotoxic lymphocytes (CTLs). The result is reduction of glioblastoma growth, associated with extensive infiltration of CD4^+^ cells and activated CD8^+^ CTLs in the TME (150). These induced cells act synergistically with PD-decoy immunotherapy and a CD-based glioblastoma vaccine with robust killing of highly resistant glioblastoma cells by tumor-specific CD8^+^ CTLs with significant improval in survival in immunocompetent animals (150). This novel approach could be used synergistically with mRNA vaccines.

Furthermore, the brain is one of the organs with the highest expression of RNA-binding proteins (RBPs); targeting the RBP complex, LOC-DHX15, with blood-brain barrier-penetrant small molecules improves treatment efficacy, impedes stem-like properties of glioblastoma cells, increases survival and offers a novel therapeutic approach to harness RNA science (151), and potentially enhance the efficacy of mRNA vaccines.

The challenges of RNA vaccines include optimization of delivery and the innate instability and immunogenicity of mRNA (152). These challenges have been largely overcome by *i*) designing modifications of the mRNA structure to avoid degradation by RNases; *ii*) optimizing purification methods to protect mRNA from contamination by double-stranded RNA to reduce nonspecific activation of the innate immune system; and *iii*) mRNA can be formulated into various nano delivery systems to deliver mRNA stably and efficiently, such as LNPs, polymers, or peptides (152). Identifying highly immunogenic, tumor-associated antigens is an inherent challenge because of individual variability; many aspects of neoepitope prediction remain to be standardized (152, 153). The large-scale production, transportation, and storage are also challenges for future applications of mRNA cancer vaccines. The speed of screening and identification of neoantigens directly affects the clinical efficacy of mRNA vaccines (153). Exploring more combinations of mRNA cancer vaccine with other therapeutic modalities is also a promising strategy (152). In view of the heterogeneity of the TME, the development of immune-based combination therapies has been a key trend in the development of cancer vaccines and in clinical trials (20–22, 153). Combinations have included the use of checkpoint inhibitors, co-stimulatory molecules (e.g., CD40), or vaccine combinations such as adoptive T cell transfer using CAR T cells (22). As a single approach, a monotherapy, is unlikely to be totally effective to eradicate a heterogeneous malignancy, especially aggressive gliomas (104), so that mRNA vaccination can be increasingly used as a “platform”, similar to the proposed use of DC vaccines (22). Additional hurdles to develop effective immunotherapies for glioblastoma center on the immunosuppressive TME, systemic immunosuppression, and immune escape mechanisms (107). These same factors pose significant challenges for the use of cellular immunotherapy for glioblastoma (129, 154) and recent advances in combination therapy for CAR T cell therapy (21) could accelerate the development of mRNA vaccines for glioblastoma and other human cancers.

Cancer cells, for example, can evolve to lose targeted antigens, thus evading the engineered CAR T cells, a phenomenon known as antigen-loss relapse (21). Efficacy can be increased by combining CAR T cell therapy with other vaccines, ICIs, oncolytic viruses, or small molecules such as ibrutinib or lenalidomide (21) that are brain penetrant (155, 156). Furthermore, ibrutinib increases survival in rodent glioma models (156); lenalidomide may help prevent T cell exhaustion (21). Within the targeted tumor, diverse cell populations add to the complexity of immunotherapeutic approaches, but recent data indicates that immune triads- a close interaction between DCs, CD4^+^ T cells, and CD8^+^ T cells, working synergistically, can dramatically eliminate solid tumors by reprogramming the CD8^+^ T cell to become functional and tumor cytolytic for a range of cancers (41). Importantly, activated T cells are uniquely able to attack dormant, disseminated cancer cells, which escape the normal immune system, standard therapy, and lead to cancer persistence, recurrence, and progression (157). If mRNA vaccines could indeed eradicate the disseminated, microscopic, minimally residual disease in glioblastoma, associated with genetic and epigenetic instability, neoplastic infiltration, oncoplasticity (104), located beyond the surgical or radiation field, it could transform the clinical outcome for patients. It appears that we have entered a new era of combined treatments (20, 21, 35). The sequencing, dosing, and timing of these multiple combinations will require well-designed clinical trials. In experimental models, combining cancer vaccines and tumor resection enables the effective infiltration of activated T cell to the resection site, with a strong impact on mouse survival (35) in an otherwise aggressive glioblastoma.

What about safety? Preliminary experience suggests that a mRNA vaccine will be relatively nontoxic (152, 153, **Table 1**). In preclinical models, a mRNA vaccine was well-tolerated: detailed toxicology in forty organs at three time points revealed no gross or microscopic findings (15). In patients with glioblastoma, a mRNA vaccine produces rapid and transient increases in pro-inflammatory cytokines, a lymphocyte nadir and neutrophilia six hours after infusion, with immune-related adverse events (e.g., low-grade fever, nausea, chills, rigors), which defervesced within 24 to 48 hours (15). These findings indicated an immunological reset with expansion and polarization of adaptive T cell responses (15). Given the early and limited experience with mRNA vaccines for human cancer, it is too early whether patients will develop cytokine release syndrome (CRS), immune effector cell-associated neurotoxicity (ICANS) or macrophage activation syndrome (MAS) which are caused by high levels of proinflammatory cytokines secreted by activated T cell and myeloid cells (21); clinical trials are exploring therapeutic interventions using antibodies such as tocilizumab for CRS and anakinra for ICANS (21). These agents, in addition to corticosteroids, would be applicable to mRNA vaccines in the event that immune-related toxicities become severe.

It is assumed that mRNA vaccines will be relatively safe because there is no integration into the DNA so the vaccine itself should not cause genomic alterations (152), as could potentially occur with plasmid-based DNA vaccines (158, 159). Furthermore, the widespread use of nucleoside-modified synthetic mRNA (*nms*-mRNA) to immunize against COVID-19 resulted in more than 782 million doses distributed to an estimated 462 million individuals by September 2022, per WHO data, and so an ongoing search for delayed safety signals remains a priority (159). There is a widespread consensus that as exogenous *“mRNA is a non-integrating platform, there is no potential risk of … insertional mutagenesis.”* (16, 159). However, a study showed that vaccine *nms*-mRNA can activate the expression of endogenous transposable elements (TEs), undergo reverse transcription and enter the cell nucleus (160), while another study showed that reverse-transcribed SARS-CoV-2 viral RNA can integrate into the genome of cultured human cell and be expressed in patient-derived tissues (161). Taken together, Acevedo-Whitehouse and Bruno hypothesized an intricate mechanism whereby the vaccine nms-mRNA, release from the LPNs into the cytosol could unsilence TE expression, enhance the expression of proinflammatory cytokines, lead to DNA damage via insertional mutagenesis and genomic instability, resulting in expression of proinflammatory cytokines (159). With the introduction of any new class of agents targeting cancer, great enthusiasm must be matched with due caution since novel interventions are frequently double-edged swords (159, 162, 163). To date, the safety signals for mRNA vaccines in clinical trials are reassuring.

## 7 Future directions

The route of delivery of mRNA, whether through an intravenous route or direct injection into tumor stands to make a difference, with some data suggesting that direct intratumor injection, “taking the fight to the tumor” (24, 26, 137, 164), could be advantageous. Local delivery of cytokine-based mRNAs can lead to a robust antitumor immune response and tumor regression in multiple tumor models (164). The cytokine-mRNA combination resulted in a ~ 50% cure rate in preclinical models of melanoma, increasing to a ~80% cure rate with the addition of ICIs, blocking metastases (164). The antitumor activity extended beyond the treated lesions and inhibited the growth of distant and disseminated tumors (164); combining mRNAs with immunomodulatory antibodies enhanced tumor regression and improved survival, leading to clinical trials of the cytokine-encoding mRNA combination (164).

As an alternative to the intratumoral release of mRNA, non-transformed cells in the liver can be exogenously transduced with mRNA in lipid formulations, thereby activating systemic biodistribution of the encoded immunostimulating factors (165). Because MHC-1 antigen presentation deficiency is a common cancer immune escape mechanism, combining tumor-targeting antibodies with IL-2 mRNA restored CD8+ T cell neoantigen immunity in MHC class I-deficient tumors that were otherwise resistant to immune-, chemo-, and radiotherapy (166). Another approach to potentiate the efficacy of mRNA vaccines would be to encode the costimulator Oxford 40 ligand, OX40L, which significantly reduces tumor growth and increases survival in preclinical models (167).

The use of small extracellular vesicles (sEVs) is a novel approach to target glioblastoma cells and generate potent antitumor activity *in vivo* (168). Using a microfluidic electroporation, which combines nano- and milli-second pulses, producing large amounts of IFN-γ mRNA-loaded sEVs with CD64 overexpressed on the surface of cells; the CD64 molecule serves as an adaptor to dock targeting ligands, such as anti-CD71 and anti-PD-L1 antibodies (168). Encapsulation of IL-12 mRNA in extracellular vesicles enables targeted delivery to treat lung cancer while promoting a systemic immune response, measured by immune memory, tumor-specific T cell priming, and expansion of tumor cytotoxic immune effector cells; IL-12 exosome-based systems could potentially be applied to other tumor types (169). RNA-loaded hydrogels have been shown to be effective *in vitro* against triple-negative breast cancer (170) and are in development for glioblastoma (32).

The use of CRISPR-Cas9 gene editing has the potential to permanently disrupt tumor survival genes, which could overcome the repeated dosing limitations of cancer therapy and improve efficacy. As proof of concept, CRISPR-Cas9 technology was applied to lipid nanoparticles containing Cas9mRNA and single-guided (sg)RNA into orthotopic glioblastoma, resulting in ~70% gene editing *in vivo*, tumor cell apoptosis, and reduction of tumor growth by 50% with improved survival by 30% (171). An elegant model of spatial manipulation of CRISPR-Cas13a activity was developed with customized RNA nanococoons featuring tumor-specific recognition and spatial-controlled activation of Cas13a and applied to suppress EGFRvIII mRNA for synergistic therapy of glioblastoma *in vitro* and *in vivo* (29).

Progress in neural networks and deep learning could be of great value to predict design of optimal antigens; high - quality, cancer neoantigen datasets could meaningfully harness the data generated by these informatic tools (172). Vaccine manufacturing will benefit from emerging solutions for the mass production of individualized vaccines, including digitization of production processes and autonomous cloud-controlled production plants fostered by advances in computational power, connectivity, human–machine interactions, robotics and innovative 3D technology enabling the building at scale of parallel, miniaturized production lines (172).

The next wave of cellular immunotherapy, including CAR T cells and dendritic cells, can take advantage of mRNA-LNP as a platform to target DCs or CD8^+^ T cells using personalized formulations incorporating neoantigens arising from genomic alterations using next-generation sequencing, immune peptidomics, and bioinformatics (173). Immune-monitoring at the single-cell or population level can be performed using peptide/MHC multimers, RNA sequencing (RNA-seq), and T cell receptor sequencing (TCR-seq) (173).

Initially, nine biotechnology startups began developing next-generation RNA drugs (174). The next wave of RNA-based drugs is using more sophisticated approaches, including tRNA to correct for errors in the genetic code that would otherwise impair protein production (174). Self-replicating RNAs, as noted (52, 53, 97) are also attractive because of their self-perpetuating, durable nature (174). Furthermore, circular RNA is more stable than its mRNA counterpart (174, 175), and there are multiple methods to produce circular RNA designed to treat glioblastoma (29). A dozen or more biotechnology firms are now pursuing therapeutics based on engineered circular RNA (circRNA), raising over US$1billion in venture capital during the past three years, betting that circRNA will emerge as the preferred RNA platform, leading to next-generation vaccines (175).

Significant challenges, however, include immunosuppressive TME, optimal candidate identification, immune response evaluation, and the need for biomarkers, as well as vaccine manufacturing acceleration (29). Undesired immunostimulation and potential impurities of the LNPs also pose a significant challenge (176). Nevertheless, the field is poised to overcome hurdles and improve patient outcomes in the future by acknowledging these clinical complexities and persistently striving to surmount inherent constraints (29). Not surprisingly, the first ARPA-H grant is centered on a mRNA platform targeting melanoma (177), hailed by President Joe Biden, urging Americans to come together for a new ‘national purpose’ (178).

## 8 Conclusion

Given the feasibility of production, the personalized approach, the minimal toxicity, and the explosion in RNA science following the success of the COVID vaccines, it is easy to predict that mRNA vaccines will be an important therapeutic option as a strategy to harness the immune system to prolong survival in patients with glioblastoma and other solid tumors. Initial results in humans using mRNA vaccines for glioblastoma are promising and support further development of mRNA vaccines as a novel approach to brain tumor therapy.

## Glossary

APC: Antigen presenting cell
circRNA: circular RNA
CNS: central nervous system
CRS: cytokine release syndrome
CSE: conserved sequence element
CTL: cytotoxic T lymphocyte
DAMP: damage-associated molecular patterns
DC: dendritic cell
GBM: glioblastoma
HNSCC: head and neck squamous cell carcinoma
HLA: human leukocyte antigen
HPV: human papilloma virus
HSV1: herpes simplex virus 1
hTERT: human telomerase reverse transcriptase
ICANS: immune effector cell-associated neurotoxicity
ICI: immune checkpoint inhibition/inhibitor
LNP: lipid nanoparticle
LPA: lipid particle aggregate
MAS: macrophage activation syndrome
MHC: major histocompatibility class
mRNA: messenger ribonucleic acid
nms-mRNA: nucleoside-modified synthetic mRNA
RBP: RNA-binding protein
RIG-I: retinoic acid-inducible gene I protein
SNV: single nucleotide variant
TCGA: The Cancer Genome Atlas
sEV: small Extracellular vesicle
TCR: T cell receptor
TE: transposable elements
TIL: tumor infiltrating lymphocyte
TLR: Toll-like receptor
TME: tumor microenvironment
TRUCK: T cells redirected for universal cytokine-mediating killing
